# Inhibition of Sphingosine Kinase 1 Reduces Sphingosine-1-Phosphate and Exacerbates Amyloid-Beta-Induced Neuronal Cell Death in Mixed-Glial-Cell Culture

**DOI:** 10.3390/neurolint16040054

**Published:** 2024-07-04

**Authors:** Tomoki Minamihata, Katsura Takano-Kawabe, Mitsuaki Moriyama

**Affiliations:** Laboratory of Integrative Physiology in Veterinary Sciences, Osaka Metropolitan University, Izumisano 598-8531, Osaka, Japan; h19950315@gmail.com (T.M.); takano@omu.ac.jp (K.T.-K.)

**Keywords:** microglia, astrocytes, sphingosine-1-phosphate, neuroinflammation

## Abstract

In Alzheimer’s disease (AD) pathology, the accumulation of amyloid-beta (Aβ), a main component of senile plaques, activates glial cells and causes neuroinflammation. Excessive neuroinflammation results in neuronal dropouts and finally produces the symptoms of AD. Recent studies suggest that disorder in sphingosine-1-phosphate (S1P) metabolism, especially the decreased expression of sphingosine kinase (SK)1, followed by the reduction in the amount of S1P, can be a promotive factor in AD onset. Thus, we explored the possibility that dysregulated S1P metabolism affects AD through the altered function in glial cells. We evaluated the effect of PF-543, a pharmacological inhibitor of SK1, on the inflammatory responses by lipopolysaccharide (LPS)-activated glial cells, microglia, and astrocytes. The treatment with PF-543 decreased the intracellular S1P content in glial cells. The PF-543 treatment enhanced the nitric oxide (NO) production in the LPS-treated neuron/glia mixed culture. Furthermore, we found that the augmented production of NO and reactive oxygen species (ROS) in the PF-543-treated astrocytes affected the microglial inflammatory responses through humoral factors in the experiment using an astrocyte-conditioned medium. The PF-543 treatment also decreased the microglial Aβ uptake and increased the number of injured neurons in the Aβ-treated neuron/glia mixed culture. These results suggest that a decrease in the glial S1P content can exacerbate neuroinflammation and neurodegeneration through altered glial cell functions.

## 1. Introduction

Alzheimer’s disease (AD) is a neurodegenerative disorder and a major causal disease of dementia. AD is mainly characterized by two pathological features: extraneuronal senile plaques in the brain and intracellular neurofibrillary tangles. It has been considered that amyloid-beta (Aβ) accumulated in brain parenchyma damage neurons and finally cause neuronal cell death, resulting in AD onset [1]. However, recent evidence suggests that other factors, such as neuroinflammation in glial cells, are involved in the onset and progression of AD [2]. In the AD brain, microglia localize near senile plaques (Aβ deposit) with an active phenotype (ameboid morphological appearance), which triggers neuroinflammatory responses, such as the induction of reactive oxygen species (ROS) production and the release of pro-inflammatory cytokines [3]. In addition, microglial activation promotes Aβ accumulation by reducing the uptake and degradation of Aβ [2]. These neuroinflammatory responses are thought to be excessive, leading to neuronal cell death and dropout, and thus the hyperactivation of microglia may cause and promote AD [2,3]. Therefore, it is important to maintain normal glial cell functioning in therapeutic strategies for AD [4].

**Scheme 1:**
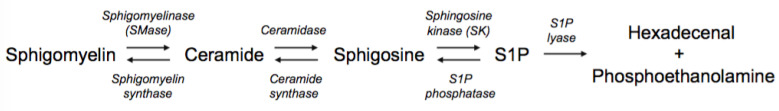
Sphingolipid metabolism.

In recent years, the metabolomic analysis of the postmortem brains of AD patients has pointed out an association between abnormal sphingolipid metabolism and AD, suggesting the possible role of disorders of lipid metabolism in the pathogenesis of AD [5,6,7,8]. In the process of sphingolipid metabolism, sphingomyelinase (SMase) catalyzes the conversion of sphingomyeline to ceramide, which is further degraded to sphingosine by ceramidase. Sphingosine kinase (SK)1/2 phosphorylates sphingosine to form sphingosine-1-phosphate (S1P), which is finally cleaved to hexadecenal and phosphoethanolamine by S1P lyase (S1PL) (see Scheme 1). The investigations of postmortem brains from AD patients have revealed that altered sphingolipid metabolism, including the decreased expression of SK1 and the increased expression of S1PL, and, consequently, decreased S1P, may promote AD pathogenesis [9]. Indeed, the downregulation of the SK1 expression increased the accumulation of Aβ with cognitive deficits in an AD model mouse [10,11]. Furthermore, decreases in the S1P levels in cerebrospinal fluid and blood have been observed early in AD patients and correlate with the progression and severity of the AD pathology [9,12]. Thus, the dynamics of S1P and its metabolizing enzymes may play a role in AD pathogenesis.

S1P, a member of the lysophospholipid family, acts as a lipid mediator. Cumulative evidence suggests that S1P is involved in inflammatory responses in various tissues, including the central nervous system (CNS) [13]. In fact, S1P induces inflammatory responses in microglia and astrocytes via S1P receptor (S1PR)-mediated signaling [14]. Moreover, the intracerebroventricular administration of lipopolysaccharide (LPS) has been reported to increase the number of activated glial cells in SK1-knockout mice compared to wild-type mice, suggesting that S1P has a detrimental effect on inflammatory responses [15]. At present, the precise roles of SK1 and S1P in glial cells during neuroinflammation remain elusive. Thus, to elucidate the role of S1P in Aβ neurotoxicity, we investigated the effect of an altered S1P metabolism induced by SK1 inhibition on glial cell functions such as nitric oxide (NO) and ROS production, Aβ uptake, and neuronal cell damage in a neuron/glia mixed culture. We confirmed that SK1 inhibition decreased the intracellular S1P contents in the glial cells, and we found that these changes exacerbated the oxidative stress (OS) and reduced the Aβ uptake by microglia, resulting in the aggravation of Aβ-induced neuronal damage. Our findings indicate that the altered S1P metabolism observed in AD, particularly the reduced S1P content, may deteriorate the disease state through alterations in glial cell functions.

## 2. Materials and Methods

### 2.1. Chemicals and Reagents

Dulbecco’s modified Eagle’s medium (DMEM), fetal bovine serum (FBS), and horse serum were obtained from Gibco BRL (Grand Island, NY, USA). LPS (derived from *E. coli*, strain 0127:B8), 2′,7′-dichlorofluorescin diacetate (H_2_DCFDA), anti-β-actin antibody, anti-Aβ_1-42_ antibody, PF-543 ((R)-(1-(4-((3-Methyl-5-(phenylsulfonylmethyl)phenoxy)methyl)benzyl)-pyrrolidin-2-yl)methanol), and K6PC-5 (2-Hexyl-N-[2-hydroxy-1-(hydroxymethyl)ethyl]-3-oxo-decanamide) were purchased from Sigma Chemical Co. (St. Louis, MO, USA). Anti-inducible NO synthase (iNOS) antibody and neuron-specific βIII-tubulin antibody were bought from R&D Systems Inc. (Minneapolis, MN, USA). Aβ_1-42_ protein (human) was purchased from the Peptide Institute, Inc. (Osaka, Japan). Fluorescence-labeled Aβ_1-42_ (f-Aβ_1-42_) was bought from Anaspec Inc. (Fremont, CA, USA). Anti-S1P antibody was purchased from Echelon Biosciences, Inc. (Salt Lake City, UT, USA). L-[2,3,4-^3^H] glutamic acid was bought from American Radiolabeled Chemicals Inc. (St. Louis, MO, USA). Forward and reverse primers for quantitative real-time polymerase chain reaction (PCR) were purchased from Eurofins Genomics Inc. (Tokyo, Japan). 2,3-Diaminonaphthalene (DAN) and 3-(4,5-dimethyl-2-thiazolyl)-2,5-diphenyl-tetrazolium bromide (MTT) were purchased from Dojindo (Kumamoto, Japan). Coomassie Brilliant Blue and protease inhibitor cocktail were bought from Nacalai Tesque (Kyoto, Japan). Horseradish peroxidase (HRP)-conjugated goat anti-mouse IgG (H+L) antibody was obtained from Bio-Rad (Hercules, CA, USA). Immobilon^TM^ Western Chemiluminescent HRP substrate was purchased from Millipore Co. (Billerica, MA, USA). Anti-glial fibrillary acidic protein (GFAP) antibody was bought from Biosensis Pty Ltd. (Thebarton, Australia). Anti-Iba-1 antibody was obtained from FUJIFILM Wako Chemicals (Osaka, Japan). FITC-conjugated goat anti-mouse IgG (H+L) and rhodamine (TRITC)-conjugated goat anti-rabbit IgG (H+L) were purchased from Jackson ImmunoResearch (West Grove, PA, USA).

### 2.2. Microglial Cultures

The mouse microglial cell line BV-2 was kindly provided by the Laboratory of Molecular Pharmacology at Kanazawa University Graduate School. BV-2 cells were maintained in DMEM supplemented with 10% FBS, 50,000 units/L penicillin, and 100 mg/L streptomycin (Sigma Chemical Co., St. Louis, MO, USA) at 37 °C in a humidified incubator under 5% CO_2_, using a dish or plate for the suspension culture (Sumilon, Sumitomo Bakelite, Co., Tokyo, Japan). Culture medium was replaced every 3–4 days, and cells were sub-cultured once a week. Preparation of rat primary microglia was carried out as previously described [16], with slight modification. In brief, whole brains of newly born 1-day-old Wistar rat pups were homogenized with DMEM by Pasteur pipette and dissociated with 0.25% trypsin in Ca^2+^- and Mg^2+^-free phosphate-buffered saline containing 5.5 mM glucose for 15 min at 37 °C in a water-bath shaker. The same quantity of horse serum supplemented with 0.1 mg/mL DNase I was added to the medium, in order to inactivate the trypsin. Then, the medium was centrifuged at 350× *g* for 5 min, and the precipitates of tissue were suspended with 10% FBS-containing DMEM supplemented with 50,000 units/L penicillin/100 mg/L streptomycin. The cells were plated onto a plastic culture flask coated with polyethyleneimine and incubated at 37 °C under 95% air/5% CO_2_ conditions. The culture medium was replaced once a week, and microglia were collected from the mixed-glial-cell culture by shaking the culture flask at 100 rpm for 1 h in 14 days in vitro. The collected cells were plated onto a 24-well plate for the suspension culture at a density of 4 × 10^5^ cells/mL. After 1 h, non-adherent cells were removed by changing the medium, and adhered microglia were maintained for 1 day in DMEM with 10% FBS. More than 95% of the cells were positive to the anti-Iba-1 antibody (rabbit polyclonal; Bio-Rad (Hercules, CA, USA)) in this culture.

### 2.3. Astrocyte Culture

Astrocytes were prepared as described previously [17]. In brief, cortices of 20-day-old Wistar rat embryos were taken out and cleared of meninges, cut into about 1 mm^3^ blocks, and treated with 0.25% trypsin in Ca^2+^- and Mg^2+^-free phosphate-buffered saline (PBS) containing 5.5 mM glucose for 20 min at 37 °C with gentle shaking. An equal volume of horse serum supplemented with 0.1 mg/mL of DNase I was added to the medium to inactivate the trypsin. Then, the tissues were centrifuged at 350× *g* for 5 min. The tissue sediments were triturated through a yellow-chip-mounted pipette with DMEM containing 10% FBS, 100 mg/L streptomycin, and 5 × 10^4^ units/L penicillin. After filtering the cell suspensions through a lens-cleaning paper (Fujifilm Co., Tokyo, Japan), the cells were plated on polyethyleneimine-coated 100 mm diameter plastic dishes (Iwaki, Asahi Glass Co., Tokyo, Japan) at a density of 0.8–1.3 × 10^5^ cells/cm^2^. Cultures were maintained in a humidified atmosphere of 5% CO_2_ and 95% air at 37 °C, changing the medium twice a week. After 1 week, astrocytes were replated to remove neurons. On days 12–14, they were replated onto adequate plates or dishes using an ordinary trypsin-treatment technique at a density of 4 × 10^5^ cells/mL for a multi-well plate (Sumitomo Bakelite Co., Tokyo, Japan; Thermo Fisher Scientific Inc., Waltham, MA, USA) or 35 mm dish (Thermo Fisher Scientific Inc., Waltham, MA, USA) and stabilized for 1 day, and then we used them for the experiments. More than 90% of the cells were immunoreactively positive to GFAP using the antibody (Biosensis) and FITC-conjugated anti-mouse IgG antibody. Less than 10% of the cells were positive to Iba-1 using the antibody (Wako) and rhodamine-conjugated anti-rabbit IgG antibody.

### 2.4. Neuron/Glia Mixed Culture

The neuron/glia mixed culture was prepared as described previously from the hippocampi of 20-day-old Wistar rat embryos in a similar manner to that described above for the astrocyte culture [17]. The cells were plated on a poly-L-lysine-coated 24-well slide glass (4 mm diameter each, Matsunami Glass Ind. Ltd., Osaka, Japan) or 8-well chamber slide glass (Nunc^TM^ Lab-Tek^TM^ II Chamber Slide^TM^ System; Thermo Fisher Scientific Inc., Waltham, MA, USA) at a density of 6.4 × 10^4^ cells/cm^2^. Cultures were maintained in a humidified atmosphere of 5% CO_2_ and 95% air at 37 °C for 1 day, and the medium was changed with DMEM containing 5% FBS. After 3 days (at 4 days in vitro), the cells were used for the experiments. The neuronal/glial mixed culture consisted of 10% neurons, 85% astrocytes, and 5% microglia as a result of the immunostaining using antibodies against a neuronal marker, βIII-tubulin (R&D systems), an astrocyte marker, GFAP (Biosensis Pty Ltd., Thebarton, Australia), and a microglial marker, Iba-1 (Wako).

### 2.5. Semi-Quantitative Measurement of Intracellular S1P Content

Intracellular S1P contents in BV-2 microglia and cultured astrocytes were measured using dot blotting via a previously published method [18]. Cells were replated onto a 35 mm dish for the suspension culture (Sumilon) or tissue culture for 24 h. Cells were treated with LPS (10 ng/mL) with PF-543 (10 or 20 μM) for 1 h. Cells were washed once with PBS and collected in a tube. After centrifugation, cell pellets were homogenized with 20 mM Tris–HCl buffer containing 5% protease inhibitor cocktail, 1 mM EDTA, 1 mM EGTA, 10 mM sodium fluoride, 10 mM sodium pyrophosphate, 10 mM β-glycerophosphoric acid, and 1 mM sodium orthovanadate. Protein concentrations were determined using BCA assay kit (Takara Bio Inc., Shiga, Japan). Protein samples were diluted to 500 ng protein/μL with buffer and frozen at −80 °C until use.

Diluted protein samples were subjected to dot blotting. A 2 μL sample (1 μg protein) was blotted on a nitrocellulose membrane and air-dried for 1 h. Then, the membrane was gently shaken in 20 mM Tris–HCl buffer containing 0.05% Tween-20 and 137 mM sodium chloride (TBST) supplemented with 5% skim milk for 1 h. Immunoblotting was conducted with primary antibodies against S1P (mouse IgG, 1:500) or β-actin (mouse IgG, 1:1000) and a secondary antibody, HRP-conjugated anti-mouse IgG antibody (1:10,000).

### 2.6. Nitrite Assay

The NO released from the cells was determined fluorometrically using the DAN reagent, as previously described [19]. BV-2 cells and cultured astrocytes were re-seeded onto 96-well plates for the suspension culture (Sumilon) or tissue culture (Thermo Fisher Scientific Inc.) for 24 h. Neuron/glia mixed culture was plated onto an 8-well chamber slide glass (Thermo Fisher Scientific Inc.) and used at 4 days in vitro. Cells were stimulated with LPS (10 ng/mL) with or without PF-543 (10 or 20 µM) for 24 h. In some experiments, the effect of dimethyl sulfoxide (DMSO) as a solvent on the NO was also investigated. The concentration of NO in the medium was determined as NO_2_^−^, a relatively stable NO metabolite, using DAN and a microplate reader (ARVO 1420 Multilabel counter, Wallac; PerkinElmer Co., Waltham, MA, USA) with excitation at 355 nm and emissions at 460 nm.

### 2.7. Cell Viability Assay

The cell viability was assessed as the total mitochondrial activity using the MTT assay, as described previously [17]. BV-2 microglia or cultured astrocytes were stimulated with LPS (10 ng/mL) in the presence or absence of PF-543 (10 or 20 µM) for 24 h. Formazan generated by incubation with the MTT solution was dissolved in DMSO and determined by measuring the optical density at 585 nm using a microplate reader.

### 2.8. Intracellular ROS Assay

The intracellular ROS generation was measured using the cell-permeable fluorescent dye H_2_DCFDA, as described previously [17]. BV-2 cells, primary microglia, and cultured astrocytes were seeded onto 96-well plates for 24 h. Cells were stimulated with LPS (10 ng/mL) with or without PF-543 (10 or 20 µM) for 24 h. Then, cells were incubated with 50 µM H_2_DCFDA in serum-free DMEM for 30 min at 37 °C. After incubation, the cells were washed once with Ca^2+^- and Mg^2+^-containing HEPES-buffered saline. The fluorescence intensity of the dichlorofluorescein was detected using a microplate reader with excitation at 485 nm and emissions at 535 nm.

### 2.9. Preparation of Astrocyte-Conditioned Medium (ACM)

Cultured astrocytes were re-seeded on a 6 cm dish (Thermo Fisher Scientific Inc., Waltham, MA, USA) for 24 h. Cells were treated with LPS (10 ng/mL) in the presence or absence of PF-543 (20 µM). After 24 h, the culture medium was collected for each stimulus group and centrifuged at 1000× *g* for 20 min, and the supernatant was used for the ACM.

### 2.10. Glutamate Uptake Assay

Cultured astrocytes were plated onto 24-well plates for tissue culture for 24 h. Cells were stimulated with LPS (10 ng/mL) with or without PF-543 (20 µM) for 24 h. After the culture medium was removed, cells were incubated with HEPES-buffered Krebs Ringer (HKR) (160 mM Na^+^, 5.4 mM K^+^, 1.8 mM Ca^2+^, 0.8 mM Mg^2+^, 5.6 mM glucose, 1 mM PO_4_^3−^, 138 mM Cl^−^, 50 mM HEPES) for 1 h. Then, the plate was placed on a copper plate at the surface of the water in a 37 °C thermostatic bath, and the HKR was removed. Reaction solution (HKR containing 10 µM glutamic acid and 20 µM [^3^H]-labeled glutamic acid) was added to each well and allowed to react for 5 min, and then the reaction was stopped by removing the reaction solution. After washing with HKR twice, cells were lysed with cell lysis solution (0.1 N NaOH containing 0.5 mg/mL SDS) for 1 h. After that, a scintillation cocktail (Clear-sol I; Nacalai Tesque, Inc., Kyoto, Japan) was added to cell lysates neutralized by 0.2 N HCl, and then the [^3^H] count per minute (cpm) was measured using a liquid scintillation counter (LSC-6100, Nippon RayTech Co., Ltd., Tokyo, Japan). Glutamate uptake by astrocytes was standardized by protein concentration of cell lysates, which was determined according to the Bradford assay [20] using Coomassie Brilliant Blue.

### 2.11. Measurement of Aβ_1-42_ Uptake

BV-2 microglia were seeded onto 96-well plates for suspension culture for 24 h. Cells were incubated with LPS (10 ng/mL) in the presence or absence of PF-543 (10 µM) for 24 h. Then, f-Aβ_1-42_, which had previously been diluted to 5 µM with culture medium and gently shaken at 37 °C overnight, was added and incubated for 4 h. Cells were washed with PBS once, and the fluorescence intensity of f-Aβ was detected using a microplate reader with excitation at 485 nm and emissions at 535 nm.

### 2.12. Preparation of Aβ_1-42_ Solution

Human Aβ_1-42_ was dissolved in DMSO to 10 mM as a stock solution and kept at −80 °C before using. Aβ_1-42_ stock solution was diluted with culture medium to 10 µM, and this was incubated for 7 days at 37 °C to form “aggregated Aβ”. For the experiments, we used it at a 100 nM Aβ_1-42_ monomer concentration.

### 2.13. Immunostaining

Immunostaining was conducted as described previously [16], with slight modification. Neuron/glia mixed culture (at 4 days in vitro) was treated with aggregated Aβ_1-42_ (100 nM) with or without PF-543 (20 μM) for 48 h. After that, the cells were fixed overnight by 4% paraformaldehyde at 4 °C. Then, cells were permeabilized with 100% methanol for 5 min at −20 °C. After blocking with PBS containing 5% bovine serum albumin (BSA) for 30 min, the cells were incubated with primary antibodies against βIII-tubulin (1:400), Iba-1 (1:400), GFAP (1:400), and Aβ (1:500) in blocking buffer at 4 °C overnight. Then, the cells were incubated with secondary antibodies against mouse IgG (FITC-labeled, 1:100) and rabbit IgG (rhodamine-labeled, 1:100) at room temperature for 1 h, followed by nuclear staining with hoechst33342 for 15 min. Cells were sealed with SlowFade^®^ Diamond (Molecular Probes). Images of the cells were captured with a confocal microscope (Olympus FV3000), and the number of each antibody-positive cell present in one field of view was counted.

Damaged neurons were assessed by hoechst33342 nuclear staining and immunostaining for βIII-tubulin, and βIII-tubulin-positive neurons with no axons or small nuclei were counted as damaged neurons and calculated as a percentage of the total neurons. Neuron-phagocytosed microglia were evaluated by double immunostaining for Iba-1 and βIII-tubulin. Neuron-phagocytosed microglia were counted as cells co-expressing Iba-1 and βIII-tubulin and Iba-1-positive cells containing βIII-tubulin positive fragments, and they were calculated as a percentage of the total number of microglia. Aβ uptake microglia were evaluated by double immunostaining for Aβ and Iba-1. Aβ uptake microglia were counted as cells co-expressing Aβ and Iba-1 and were calculated as a percentage of the total number of microglia.

### 2.14. Real-Time Quantitative Polymerase Chain Reaction (PCR)

BV-2 cells were seeded onto 24-well plates for suspension culture for 24 h. Cells were treated with aggregated Aβ_1-42_ (1 μM) with or without PF-543 (10 μM) for 24 h. Total RNA was extracted from cells using the FavorPrep^TM^ Tissue Total RNA Purification Mini Kit (Favorgen, Taiwan), according to the manufacturer’s protocol. Complementary DNA was synthesized from the extracted RNA using the Omniscript Reverse Transcription Kit (Qiagen, Germany), according to the manufacturer’s protocol. Real-time quantitative PCR was performed using SYBR^®^ Green Realtime PCR Master Mix (Toyobo Co., Ltd., Osaka, Japan) and a real-time PCR system (StepOne^TM^; AppliedBiosystems, Thermo Fisher Scientific Inc.). The list of the primer sequences (Eurofins Genomics Inc., Tokyo, Japan) and cycling protocols used in this study are shown in Table 1. Fluorescent products were detected at the end of the elongation period. To compare mRNA levels across samples, the expression of mRNA for each gene of interest was normalized against that for a housekeeping gene 18S rRNA using the comparative Ct method. The alteration in the levels of each PCR product was calculated as a ratio of the control.

### 2.15. Statistical Analysis

Data are presented as the means ± standard errors. For statistical analysis of the data, Tukey’s multiple-comparison procedure following a one-way analysis of variance (ANOVA) or Student’s *t* test was employed. The results were considered statistically significant at *p* < 0.05.

## 3. Results

### 3.1. Effect of SK1 Inhibitor on Intracellular S1P Content in LPS-Treated Glial Cells

**Figure 1:**
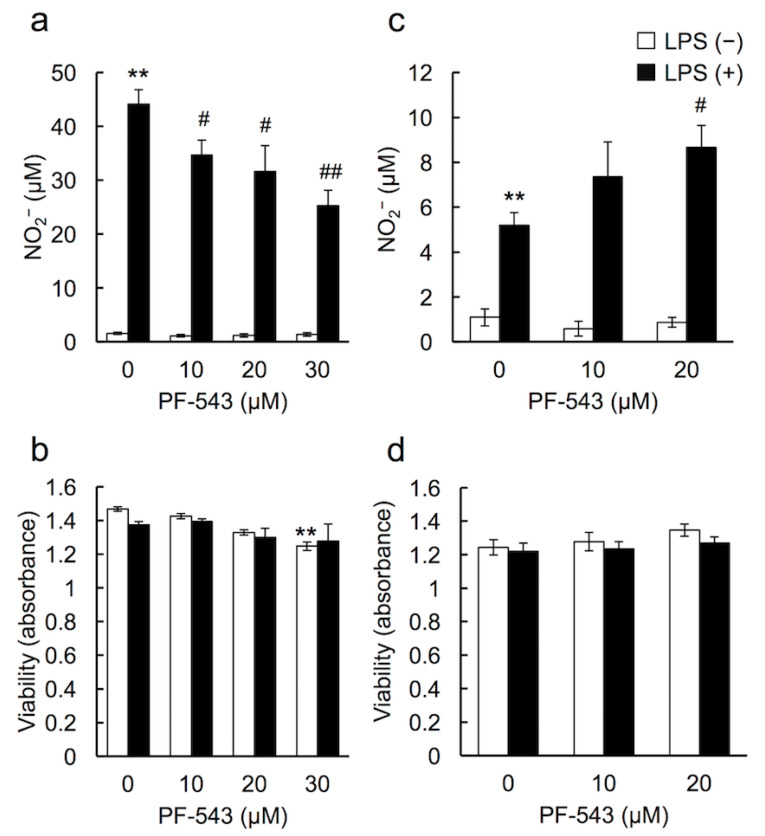
Effect of PF-543 on NO production by LPS-treated BV-2 microglia and primary astrocytes. Cells were treated with 10 ng/mL LPS together with various concentrations (10, 20, 30 μM, (**a**,**b**); BV-2, or 10, 20 μM, (**c**,**d**); astrocytes) of PF-543 for 24 h. Nitrite concentrations in the medium were measured using a fluorescence assay with the DAN reagent (**a**,**c**). Cell viability was evaluated using the MTT assay (**b**,**d**). Data are means ± standard errors of 4–9 samples. ** *p* < 0.01, significantly different from control; ^#^ *p* < 0.05, ^##^ *p* < 0.01, significantly different from LPS by one-way ANOVA followed by Tukey’s multiple-comparison procedure.

Previous reports have demonstrated that the treatment of the SK1 inhibitor PF-543 decreased the intracellular S1P contents in several types of cells, including head and neck tumor cells [21]. Therefore, we first examined the efficacy of PF-543 on the S1P content in LPS-stimulated BV-2 microglia and astrocytes. In our preliminary experiments, we applied PF-543 to BV-2 cells (and astrocytes) at several concentration ranges, according to previous reports [21]. Since the treatment of 30 μM of PF-543 decreased the cell viability in BV-2 cells and that of 10 μM was sufficiently effective for NO production, which is representative of the inflammatory responses in microglia, (Figure 1a,b), we used PF-543 at 10 μM in microglial cells for further study. In astrocytes, the treatment of 10 μM of PF-543 had no effect on the NO production, but that of 20 μM significantly increased it without affecting cell viability (Figure 1c,d). Therefore, 20 μM of PF-543 was employed in the astrocytes in all the subsequent experiments. 

**Figure 2:**
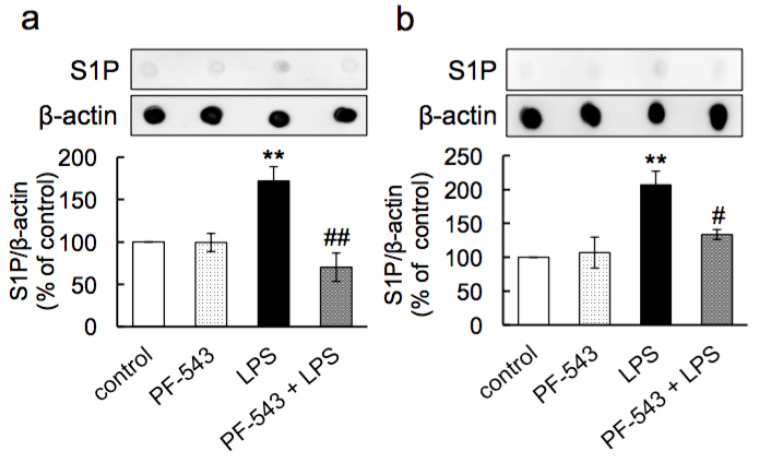
Effects of PF-543 on intracellular S1P content in LPS-stimulated BV-2 microglia and primary astrocytes. Cells were treated with 10 ng/mL LPS with or without 10 ((**a**); BV-2) or 20 ((**b**); astrocytes) μM of PF-543 for 1 h. Intracellular S1P content was measured by dot blot assay using anti-S1P antibody. Typical dots for S1P and β-actin protein are shown in the photograph. The graph shows S1P/β–actin ratio of the density of detection dots. Data are shown relative to control. Data are means ± standard errors of 4 (**a**) or 4–5 (**b**) samples. ** *p* < 0.01, significantly different from control; ^##^ *p* < 0.01, ^#^ *p* < 0.05, significantly different from LPS by one-way ANOVA followed by Tukey’s multiple-comparison procedure.

The cells were treated with 10 ng/mL of LPS in the presence or absence of 10 or 20 μM of PF-543 for 1 h, and the intracellular S1P amount was measured by dot blot assay using anti-S1P antibody. The LPS treatment markedly increased the intracellular S1P content both in the BV-2 microglia and astrocytes (Figure 2). The treatment with PF-543 significantly decreased the intracellular S1P content in the LPS-treated BV-2 cells and astrocytes. These results confirmed that the SK1 inhibition by PF-543 decreases in the intracellular S1P content in glial cells.

### 3.2. Decrease in Glial S1P Content Augmented NO Production in LPS-Treated Neuron/Glia Mixed Culture

**Figure 3:**
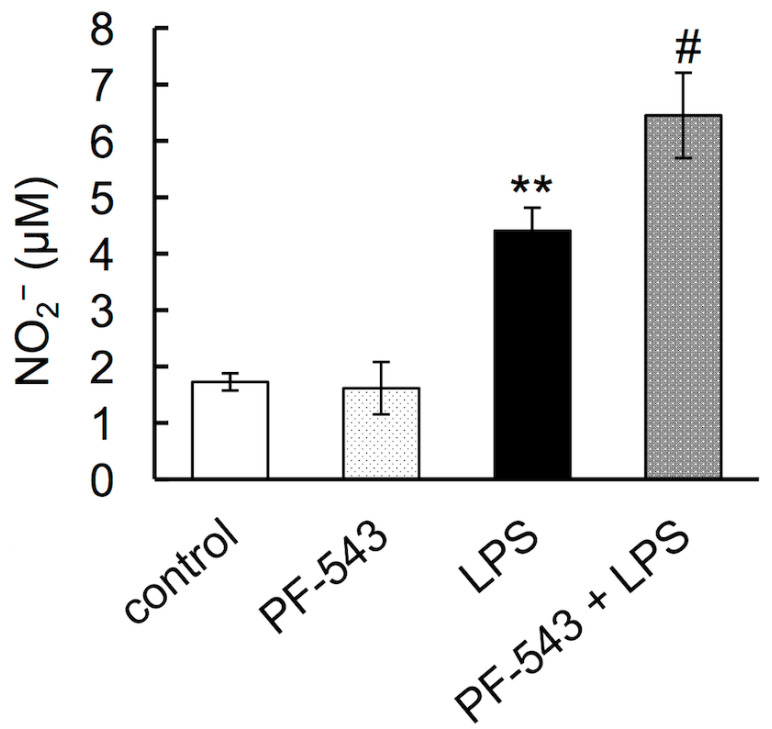
Effect of PF-543 on NO production in LPS-treated neuron/glia mixed culture. Neuron/glia mixed culture was treated with 10 ng/mL LPS in the presence or absence of 20 µM PF-543 for 24 h. Nitrite concentrations in the medium were measured using a fluorescence assay with the DAN reagent. Data are means ± standard errors of 4–5 samples. ** *p* < 0.01, significantly different from control; ^#^ *p* < 0.05, significantly different from LPS by one-way ANOVA followed by Tukey’s multiple-comparison procedure.

The activation of glial cells leads to excessive inflammatory responses, such as increases in the production of NO and pro-inflammatory cytokines and the generation of ROS, resulting in neuroinflammation in the brains of AD patients [2,3]. Thus, we first assessed the impact of the reduction in the intracellular S1P content by SK1 blockade on the NO production by glial cells. The neuron/glia mixed culture was stimulated with 10 ng/mL of LPS in the presence or absence of 20 μM of PF-543 for 24 h, and the NO production in the medium was analyzed. The LPS treatment significantly increased the NO production in the neuron/glia mixed culture (Figure 3). Although the PF-543 treatment did not affect the NO production without LPS stimulation, the co-treatment of PF-543 significantly increased the NO production in the LPS-treated neuron/glia mixed culture. 

**Figure 4:**
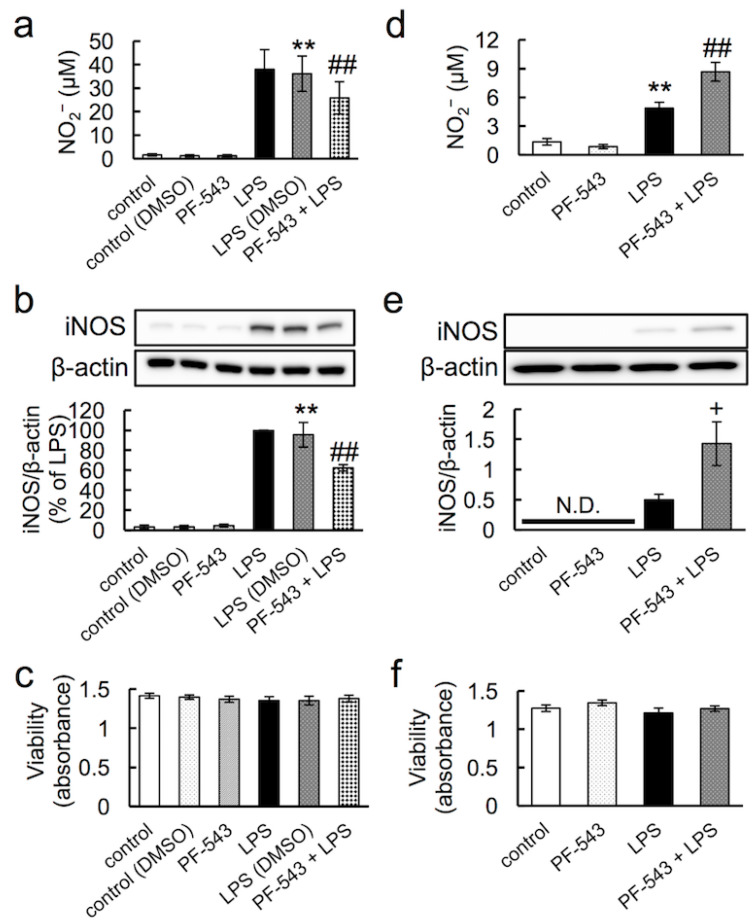
Effect of PF-543 on NO production by LPS-treated BV-2 microglia and primary astrocytes. Cells were treated with 10 ng/mL LPS with or without 10 ((**a**–**c**); BV-2) or 20 ((**d**–**f**); astrocytes) μM PF-543 for 24 h. Nitrite concentrations in the medium were measured using a fluorescence assay with the DAN reagent (**a**,**d**). Cell viability was evaluated using the MTT assay (**c**,**f**). Expression of iNOS protein was detected by immunoblotting (**b**,**e**). Typical bands for iNOS and β-actin protein are shown in the photograph. The graph shows iNOS/β–actin ratio of the density of detection bands. Data are shown relative to 10 ng LPS/mL in the immunoblotting analysis for BV-2 cells (**d**). Data are means ± standard errors of 4 samples. ** *p* < 0.01, significantly different from control (DMSO) (**a**,**b**) or control (**d**); ^##^ *p* < 0.01, significantly different from LPS (DMSO) (**a**,**b**) or LPS (**d**) by one-way ANOVA followed by Tukey’s multiple-comparison procedure; ^+^ *p* < 0.05, significantly different from LPS (**f**) by Student’s *t* test.

To explore which glial cells are responsible for the augmented NO production, we investigated the effect of PF-543 on the NO production in each glial cell, microglia, and astrocyte. The treatment of PF-543 significantly suppressed the NO production in the LPS-treated BV-2 microglia (Figure 4a). The PF-543 treatment decreased the inducible NO synthase (iNOS) protein expression in the LPS-stimulated BV-2 cells as well as the NO production (Figure 4b).

We also conducted similar experiments using astrocytes. Contrary to the results for the microglia, the PF-543 treatment remarkably increased both the NO production and iNOS protein expression in the LPS-stimulated astrocytes (Figure 4d,e). The treatment of LPS with PF-543 used in this study did not show any significant effect on the cell viability of either the microglia or astrocytes (Figure 4c,f). From these results, the augmented NO production observed in the neuron/glia mixed culture by PF-543 might reflect its effect on astrocytes.

**Figure 5:**
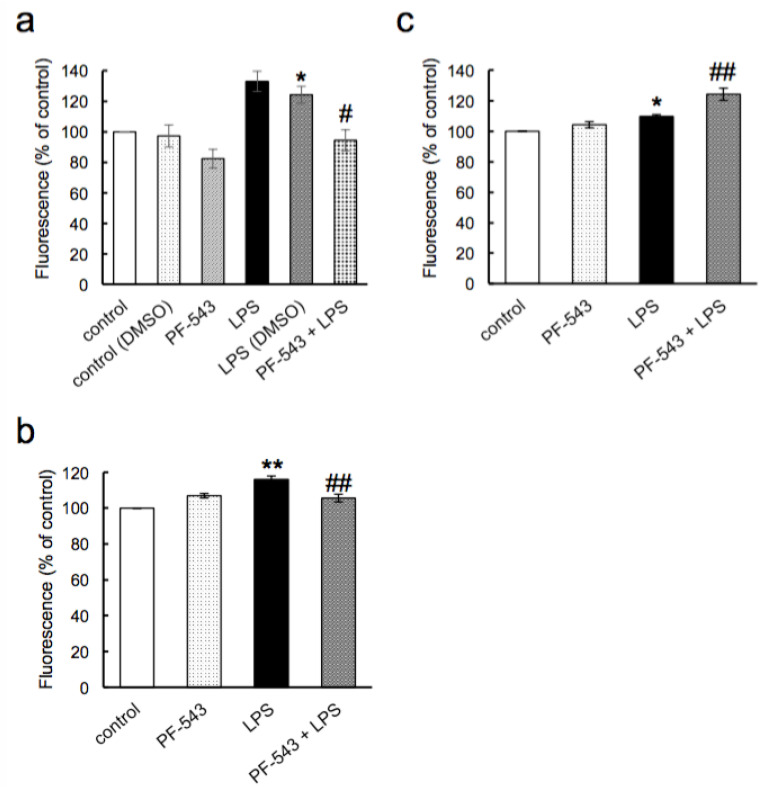
Effect of PF-543 on ROS generation in LPS-treated microglia and astrocytes. Cells were treated with 10 ng/mL LPS in the presence or absence of 10 ((**a**); BV-2, (**b**); primary microglia) or 20 ((**c**); astrocytes) μM PF-543 for 24 h. Intracellular ROS generation was determined using H_2_DCFDA, a cell-permeable fluorescent dye. Data are shown relative to the control group. Data are mean ± standard errors of 4 (**a**), 3 (**b**), or 5 (**c**) samples. ** *p* < 0.01, * *p* < 0.05, significantly different from control (DMSO) (**a**) or control (**b**,**c**); ^##^ *p* < 0.01, ^#^ *p* < 0.05, significantly different from LPS (DMSO) (**a**) or LPS (**b**,**c**) by one-way ANOVA followed by Tukey’s multiple-comparison procedure.

Next, we further elucidated the effect of PF-543 on the generation of intracellular ROS, the OS marker. Similar to the NO production, the treatment of PF-543 significantly decreased the LPS-induced ROS generation in the BV-2 microglia (Figure 5a), but it markedly increased the ROS production in the astrocytes (Figure 5c). We also conducted similar experiments using primary microglia to confirm whether the same phenomenon observed in the BV-2 cells can be reproduced. The treatment of PF-543 reduced the LPS-induced ROS generation in the primary microglia (Figure 5b). These results suggest that the decrease in cellular S1P by PF-543 ameliorates neuroinflammatory responses such as the NO and ROS production in activated microglia, but not in astrocytes.

**Figure 6:**
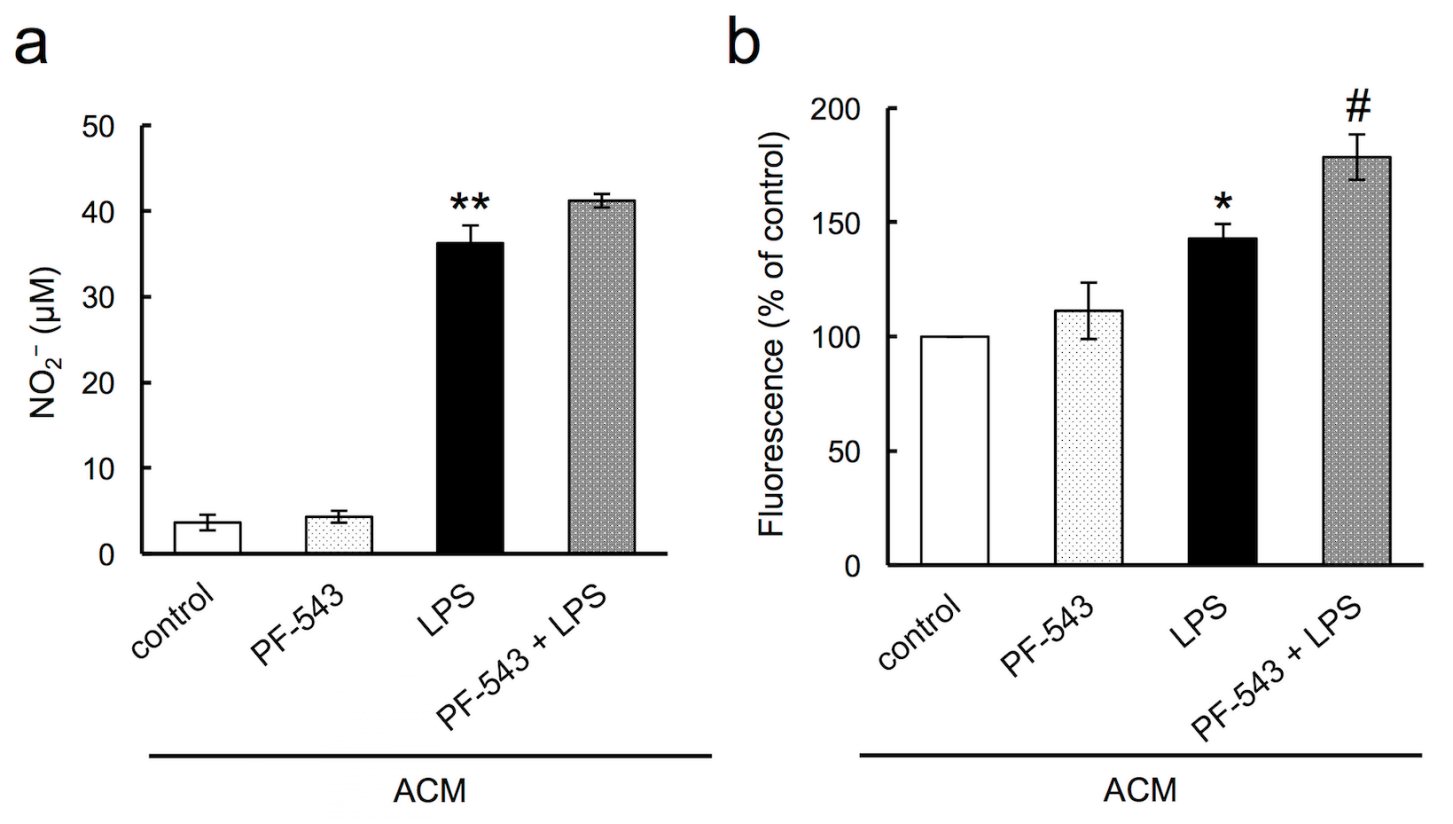
Effect of astrocyte-conditioned medium from LPS- and PF-543-treated astrocytes on NO and ROS production in BV-2 microglia. Astrocyte-conditioned medium (ACM) was prepared from cultured astrocytes treated with 10 ng/mL LPS with or without 20 μM PF-543 for 24 h. BV-2 cells were incubated with ACM for 24 h. Nitrite concentrations in the medium were measured using a fluorescence assay with the DAN reagent (**a**). Intracellular ROS generation was determined using H_2_DCFDA, a cell-permeable fluorescent dye (**b**). Data are shown relative to the control group in the ROS assay. Data are mean ± standard errors of 4 (**a**) or 5 (**b**) samples. ** *p* < 0.01, * *p* < 0.05, significantly different from control; ^#^ *p* < 0.05, significantly different from LPS by one-way ANOVA followed by Tukey’s multiple-comparison procedure.

To elucidate the possible mechanism of PF-543 in the augmented NO production in neuron/glia cells, the interaction between the microglia and astrocytes was analyzed. We examined the effect of the astrocyte-conditioned medium (ACM) on microglial inflammatory responses such as the NO production and ROS generation. The ACM was prepared from astrocytes that were stimulated with 10 ng/mL of LPS with or without 20 µM of PF-543 for 24 h. The treatment with the LPS-stimulated ACM significantly increased both the NO production (Figure 6a) and ROS generation (Figure 6b) in the BV-2 microglia. The ACM obtained from the co-treatment with PF-543 and LPS significantly increased the ROS level and had a tendency to increase the NO production compared to the LPS-stimulated ACM. Based on these results, we considered that the augmented inflammatory responses, especially the OS response by astrocytes, can spill over into microglial inflammation, resulting in exacerbated neuroinflammatory responses in the condition in which both neurons and glial cells are present.

### 3.3. Decrease in S1P Content Reduced Glutamate Uptake by Astrocytes

**Figure 7:**
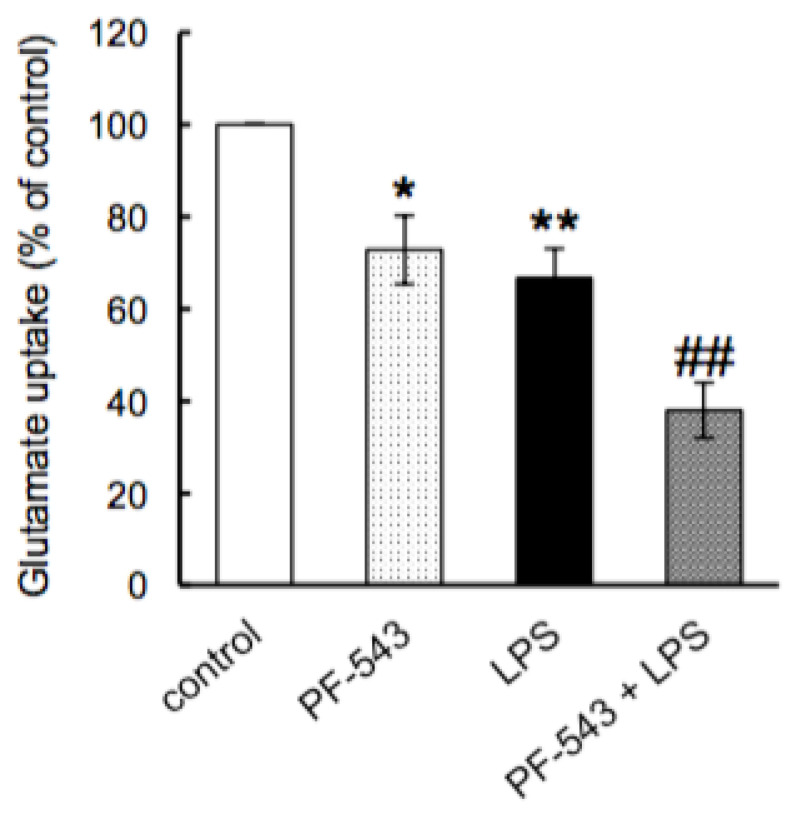
Effect of PF-543 on glutamate uptake by astrocytes. Primary astrocytes were treated with 10 ng/mL LPS in the presence or absence of 20 μM PF-543 for 24 h. Glutamate uptake was assessed using [^3^H]-conjugated glutamate and liquid scintillation counter. Data are shown relative to the control group. Data are mean ± standard errors of 7 samples. ** *p* < 0.01, * *p* < 0.05, significantly different from control; ^##^ *p* < 0.01, significantly different from LPS by one-way ANOVA followed by Tukey’s multiple-comparison procedure.

The glutamate uptake by astrocytes is essential to protect neurons from glutamate-induced excitotoxicity by maintaining low extracellular glutamate concentrations [22]. Since Aβ increases the extracellular glutamate concentrations and causes excitatory neurotoxicity via NMDA receptors in AD [23], the glutamate uptake by astrocytes is also important for AD. Thus, we investigated whether decreases in the S1P content by PF-543 affect the glutamate uptake by astrocytes. Cultured astrocytes were stimulated with LPS in the presence or absence PF-543 for 24 h, and the glutamate uptake was determined by measuring the intracellular glutamate concentration using [^3^H]-conjugated glutamate. Although the addition of PF-543 or LPS significantly decreased the astrocytic glutamate uptake, the co-treatment of PF-543 with LPS further reduced it compared to each treatment alone (Figure 7). These results suggest that the decreased cellular S1P content induced by PF-543 in astrocytes may abbreviate the synaptic glutamate clearance.

### 3.4. Decrease in Microglial S1P Content Reduced Aβ uptake in Aβ-Treated Neuron/Glia Mixed Culture

**Figure 8:**
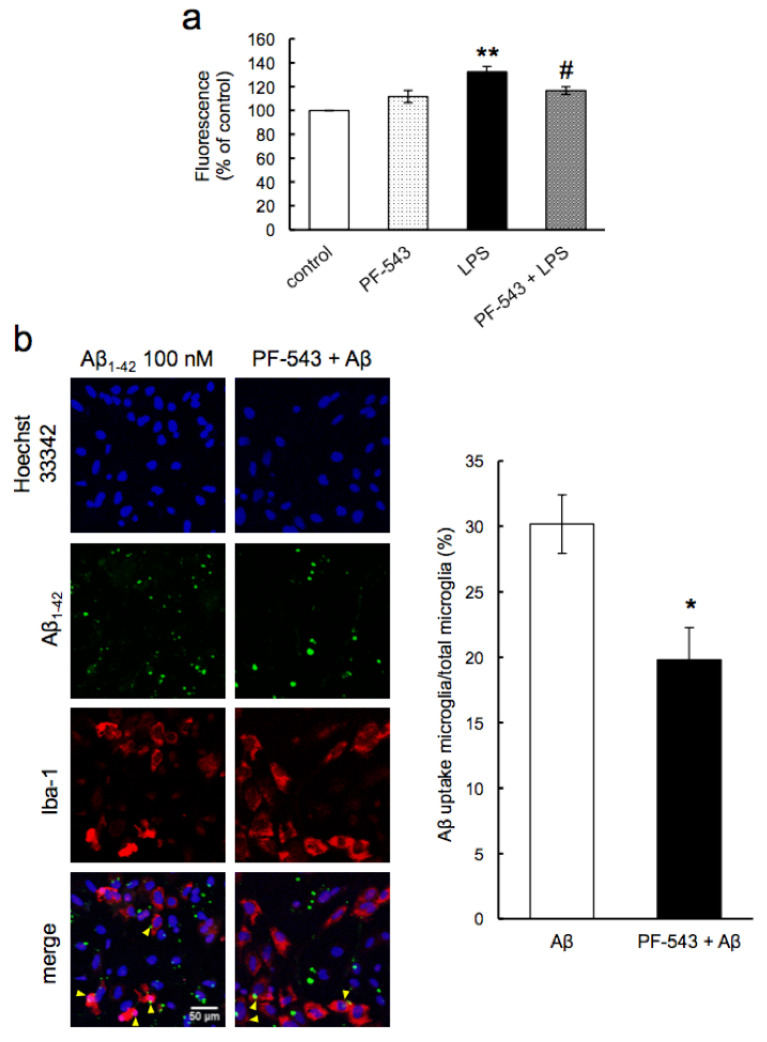
Effect of PF-543 on Aβ uptake by microglia. (**a**) Primary microglia were treated with 10 ng/mL LPS with or without 10 μM PF-543 for 24 h. Aβ uptake by cells was measured using fluorescence-labeled Aβ_1-42_. (**b**) Neuron/glia mixed culture was incubated with pre-aggregated Aβ_1-42_ (100 nM at monomer concentration) in the presence or absence of 20 μM PF-543 for 48 h. The number of Aβ uptake microglia was counted after immunocytochemical staining. Arrowhead means microglia that engulfed Aβ. Data are shown relative to the control group (**a**), or as the ratio of the Aβ uptake microglia against the total microglia (**b**). Data are mean ± standard errors of 4 (**a**) or 3 (**b**) samples. (**a**) ** *p* < 0.01, significantly different from control; ^#^ *p* < 0.05, significantly different from LPS by one-way ANOVA followed by Tukey’s multiple-comparison procedure. (**b**) * *p* < 0.05, significantly different from Aβ by Student’s *t* test. Scale bar = 50 µm.

In AD, the accumulation and aggregation of Aβ in the brain cause the death and loss of neurons [23]. The uptake and degradation of accumulated Aβ is one of the important microglial functions to the maintenance of a normal CNS environment, and the decreased clearance of Aβ by microglia is crucial for the onset and progression of AD [23]. Therefore, we assessed the effect of a decreased cellular S1P content (by PF-543) on the microglial Aβ uptake using f-Aβ. First, primary microglia were stimulated with 10 ng/mL of LPS in the presence or absence of 10 μM of PF-543 for 24 h, and then f-Aβ_1-42_ was added. The addition of PF-543 did not have any significant effect on the uptake of f-Aβ_1-__42_ (Figure 8a). The LPS treatment increased the uptake of f-Aβ_1-42_; however, the co-treatment with PF-543 suppressed the LPS-induced uptake of f-Aβ_1-42_. Next, to confirm that the PF-543 treatment affected the microglial Aβ uptake in the neuron/glia mixed culture, we determined the number of microglia that engulfed Aβ_1-42_. Aβ_1-42_ was aggregated by incubation for 7 days at 37 °C, and then the neuron/glia mixed culture was treated with pre-aggregated Aβ_1-42_ in the presence or absence of PF-543 for 48 h. In the absence of PF-543, about 30% of microglia phagocytose aggregated Aβ_1-42_ (Figure 8b). By contrast, the microglial phagocytosis of Aβ_1-42_ was significantly decreased in the presence of PF-543. These results indicate that decreases in the cellular S1P content (by PF-543) impair the clearance of Aβ by microglia, which may accelerate the Aβ accumulation.

### 3.5. Decrease in S1P Content Exacerbated Neuronal Damage and Increased Microglial Phagocytosis of Neurons in Aβ-Treated Neuron/Glia Mixed Culture

**Figure 9:**
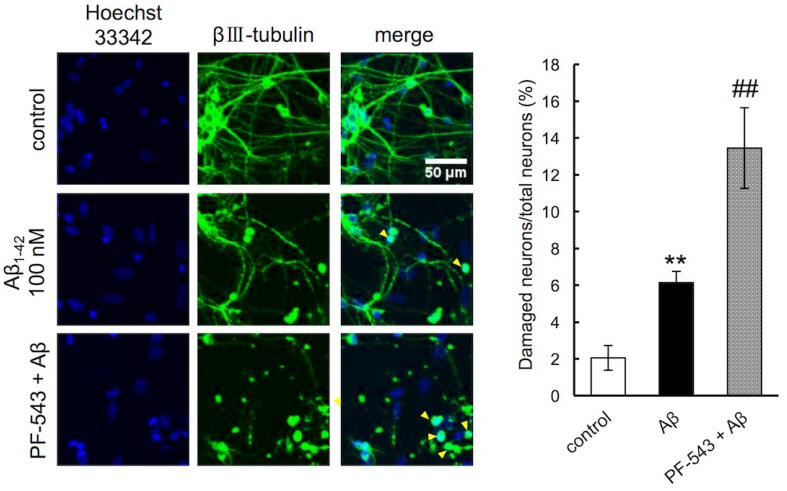
Effect of PF-543 on neuronal damage in neuron/glia mixed culture. Neuron/glia mixed culture was treated with pre-aggregated Aβ_1-42_ (100 nM at monomer concentration) in the presence or absence of 20 μM PF-543 for 48 h. The number of damaged neurons (arrowhead) that had no axons was counted after immunocytochemical staining. Data are shown as the ratio of damaged neurons against total neurons. Data are mean ± standard errors of 4–7 samples. ** *p* < 0.01, significantly different from control; ^##^ *p* < 0.01, significantly different from Aβ by a Student’s *t* test. Scale bar = 50 µm.

From the results obtained in this study, a decrease in S1P by PF-543 hampers the microglial clearance of Aβ. Aβ triggers inflammatory responses by glial cells via cell surface receptors, such as Toll-like receptors (TLRs), leading to neuronal cell death [2,3]. Thus, the decrease in the Aβ uptake by microglia may exacerbate the Aβ-induced neuronal damage when neurons and glial cells co-exist, which is close to the environment in the brain. To test this, we explored the effects of PF-543 on the neuronal damage in the neuron/glia mixed culture. The number of damaged neurons, which have no axons, was increased by the treatment with pre-aggregated Aβ_1-42_ (Figure 9). The co-treatment with PF-543 and Aβ_1-42_ further augmented the number of damaged neurons.

**Figure 10:**
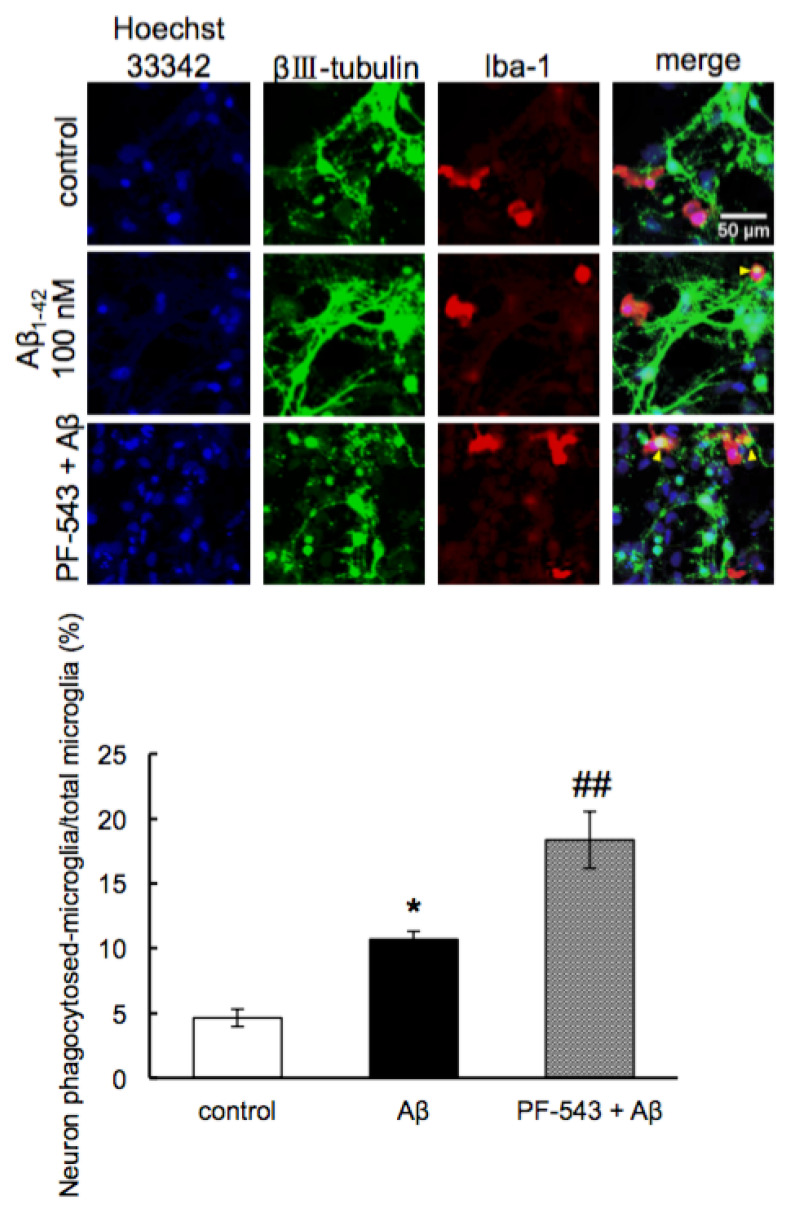
Effect of PF-543 on microglial phagocytosis of neurons in neuron/glia mixed culture. Neuron/glia mixed culture was treated with pre-aggregated Aβ_1-42_ (100 nM at monomer concentration) with or without 20 μM PF-543 for 48 h. The number of microglia that took in neurons or debris (arrowhead) was counted after immunocytochemical staining. Data are shown as the ratio of neuron-phagocytosed microglia against total microglia. Data are mean ± standard errors of 5–8 samples. * *p* < 0.05, significantly different from control; ^##^ *p* < 0.01, significantly different from Aβ by one-way ANOVA followed by Tukey’s multiple-comparison procedure. Scale bar = 50 µm.

In addition, the effect of PF-543 on the microglial phagocytosis of neurons was also investigated in the neuron/glia mixed culture. Similar experiments were also conducted, and the microglia that phagocytosed damaged neurons was evaluated. Aβ_1-42_ increased the microglia that phagocytosed injured neurons (Figure 10). This was further enhanced in the presence of PF-543. Collectively, these results indicate that a decrease in glial S1P aggravates Aβ-induced neuronal damage and finally results in neuronal loss by the enhanced microglial phagocytosis of cell debris.

### 3.6. Decrease in S1P Content Reduced mRNA Expressiosn of PPAR-γ and CD36 and Molecules Associated with Aβ Uptake in BV-2 Microglia

**Figure 11:**
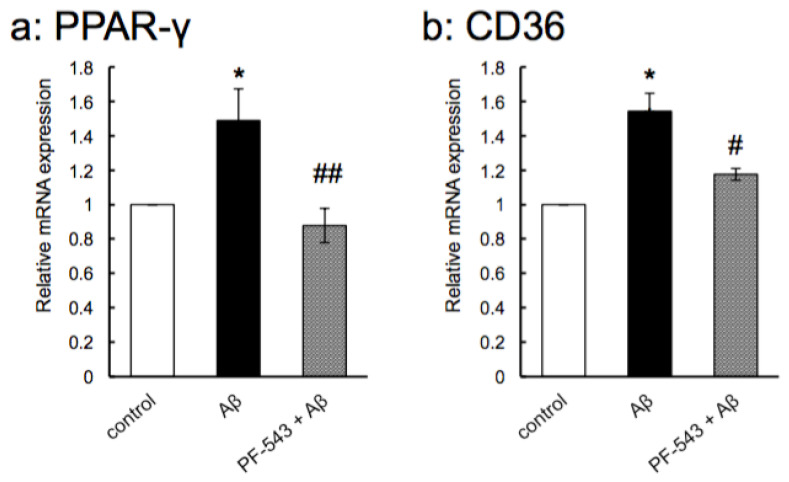
Effect of PF-543 on mRNA expression of PPAR-γ and CD36 in BV-2 microglia. Cells were treated with pre-aggregated Aβ_1-42_ (1 μM at monomer concentration) with or without 20 μM PF-543 for 24 h. The level of mRNA expression was determined by real-time quantitative PCR with the delta–delta Ct method. Data are shown relative to the control group. Data are mean ± standard errors of 6 (**a**) or 3 (**b**) samples. * *p* < 0.05, significantly different from control; ^##^ *p* < 0.01, ^#^ *p* < 0.05 significantly different from Aβ by one-way ANOVA followed by Tukey’s multiple-comparison procedure.

Numerous studies have demonstrated that microglia take up Aβ via several receptors [23]. Among them is the microglia phagocytose of Aβ via CD36, one of the scavenger receptors, and this is mediated by PPAR-γ signaling [23,24,25]. Moreover, previous reports have revealed that intracellular S1P directly interacts with PPAR-γ and activates it as a ligand [26]. Next, to obtain a mechanistic insight into the contribution of PF-543 to the Aβ uptake, we investigated the effect of PF-543 on the mRNA expression of PPAR-γ and CD36 in the Aβ-treated BV-2 microglia. We treated BV-2 cells with 1 μM of aggregated Aβ_1-42_ in the presence or absence of 10 μM of PF-543 for 24 h, and we then determined the mRNA expression by real-time quantitative PCR. The treatment with Aβ_1-42_ enhanced the mRNA expression of both PPAR-γ and CD36 in the BV-2 microglia (Figure 11). The addition of PF-543 alone had no effect on the mRNA expression of either PPAR-γ or CD36. The treatment with PF-543 completely suppressed the Aβ_1-42_-induced increase in the mRNA expression of both PPAR-γ and CD36. These results suggest that a decrease in the intracellular S1P level by PF-543 may suppress the microglial Aβ uptake through the downregulation of the PPAR-γ and CD36 signaling pathways.

## 4. Discussion

Several lines of evidence document that the decrease in the amount of intracellular S1P is accompanied by reduced SK1 protein expression in postmortem AD brains [9]. Furthermore, SK1 knockdown by siRNA in a mouse model of AD exacerbated the loss of the memory and learning abilities and Aβ accumulation in the brain [10,11]. These findings suggest that the decreased S1P level and the expression of SK1 can play key roles in AD pathology. However, the mechanism behind how an altered S1P metabolism affects pathologic events in AD, including Aβ accumulation and neuroinflammation, is not clear. Thus, we examined the effects of a decrease in S1P on the functions of glial cells that play an important part in Aβ clearance and neuronal damage. Our study demonstrated that the treatment with PF-543, an SK1 inhibitor, augmented the LPS-induced neuroinflammatory responses, and it especially increased the OS responses by glial cells and reduced the Aβ clearance by microglia, resulting in exacerbated neuronal damage in the Aβ-treated neuron/glia mixed culture. Therefore, our findings support the possibility that an abnormal S1P metabolism in glial cells may play a pivotal role in the mechanism of AD pathogenesis.

Our study demonstrated that an SK1 blockade by PF-543 increased the NO production in the neuron/glia mixed culture (Figure 3). Using a mono-cultured system, PF-543 augmented the oxidative stress response, including the NO and ROS generation in the astrocytes (Figure 4d and Figure 5c), although that of the microglia was suppressed (Figure 4a and Figure 5a,b). To fill in the gap between the action of PF-543 in astrocytes and microglia, the effect of the ACM was evaluated, revealing that the treatment of the ACM obtained from the co-treatment with PF-543 and LPS significantly increased the ROS and tended to increase the NO production compared to the LPS-stimulated ACM in the BV-2 microglia (Figure 6a,b). These results suggest that humoral factors derived from astrocytes may counteract the inhibitory effect of PF-543 on the NO and ROS production observed in mono-cultured microglia. The details of the humoral factors responsible for this counteraction were not identified in the present study; however, it is possible that the NO and ROS produced by astrocytes may act directly on microglia. Stimulation with H_2_O_2_, a well-known inducer of OS, triggers the activation of mitogen-activated protein kinase (MAPK) and nuclear factor-kappa B (NF-κB), signaling pathways that stimulate the production of NO and ROS in microglia [27,28]. Thus, ROS, including NO derived from astrocytes, might act on microglia and exacerbate the microglial oxidative stress response. Additional experiments are necessary to identify the humoral factors from astrocytes under neuroinflammation.

Our present study showed that the decrease in the intracellular S1P level by SK1 inhibition impaired the microglial clearance of Aβ (Figure 8b). Recent reports have revealed the mechanisms of Aβ uptake by microglia, including the Aβ receptor-mediated pathway [23,29,30]. Among them, we focused on PPAR-γ and CD36 as receptor-mediated mechanisms because intracellular S1P interacts with PPAR-γ and affects its activity [26]. Microglia have been shown to take up Aβ via CD36, one of the scavenger receptors [23,25]. In addition, Yamanaka and colleagues [24] have reported that PPAR-γ upregulates the CD36 expression, stimulating Aβ phagocytosis in microglia. Our study demonstrated that the SK1 blockade reduced the Aβ_1-42_-induced mRNA induction of both PPAR-γ and CD36 (Figure 11). Therefore, the modulation of the CD36 expression by PPAR-γ may account for the suppression of the Aβ uptake by altered intracellular levels of S1P. Further studies are necessary to verify this possibility by examining the effects of PPAR-γ agonists or antagonists on PF-543-treated cells.

Astrocytes express S1PR_1-3_ on the cell membrane surface [14]. Extracellularly applied S1P induces inflammatory responses, such as pro-inflammatory cytokine production via S1PR in astrocytes [31,32]. In the present study, the reduction in cellular S1P augmented the LPS-induced NO and ROS production in astrocytes (Figure 4d and Figure 5c), suggesting that the intracellular S1P acted as an anti-inflammatory agent. The reason for the differential effect of S1P on inflammatory responses is unclear but may be due to the difference in its location, namely, extracellularly applied or intracellularly synthesized S1P. Additionally, the presence of S1P transporters, like spinster homolog 2 (Spns2), which transports intracellular S1P across the cell membrane, has not been reported previously in astrocytes. Thus, the intracellular S1P modulation of inflammatory responses through the S1PR signaling pathway can be ruled out. Another possible explanation is the involvement of PPAR-γ. It has been shown that free S1P in the cytoplasm directly binds to PPAR-γ, stimulating the transcription of PPAR-γ target genes [26]. A PPAR-γ agonist reduced the ROS generation in CTW-TNA2 cells, a rat astrocyte cell line [33]. In addition, a PPAR-γ antagonist reduced the expression and activity of catalase, one of the major enzymes for ROS degradation, in astrocyte peroxisomes [33,34]. These results collectively suggest that PPAR-γ activation leads to the suppression of inflammatory responses in astrocytes. Although we did not measure the expression of PPAR-γ in astrocytes, PPAR-γ signaling may be an important factor in neuroinflammation.

Although our current study demonstrated that the treatment of PF-543 exacerbated the neuronal damage induced by aggregated Aβ in the neuron/glia mixed cultures (Figure 9), how the decrease in endogenous S1P affects the neuronal function is unclear. It is possible that S1P acts as an anti-apoptotic molecule. Various studies indicate that the decrease in endogenous S1P induces neuronal cell death in the brains of AD patients [9,10,11]. However, we observed that the reduction in the intracellular S1P content did not affect the cell viability of neuronal cells, SH-SY5Y human neuroblastoma cells (Supplementary Figure S1). These results collectively suggest that the alterations in the glial functions contribute to neuronal damage induced by the decrease in the intracellular S1P. Indeed, our present study showed that the decrease in endogenous S1P impeded the ability of astrocytes to uptake glutamate (Figure 7). The mechanism by which the decrease in S1P inhibits glutamate uptake is not clear. The knockdown of S1PR has been reported to decrease GLT-1 mRNA expression [35], suggesting that S1P-S1PR signaling may be necessary for astrocytic glutamate reuptake. Moreover, it has been reported that ROS inhibits the glutamate uptake in astrocytes [36], and the LPS-induced increase in ROS production (Figure 5c) might further decrease the glutamate uptake under conditions of low S1P levels. Consistent with this, a high concentration of extracellular glutamate causes excitatory neurotoxicity via NMDA receptors in AD brains [23]. Therefore, it is possible that the amount of intracellular S1P is a key factor in the protection from excitatory neurotoxicity in AD brains. In support of this mechanism, a recent study demonstrated that the increase in endogenous S1P attenuates Aβ-induced neuronal death by the endocytic internalization of extrasynaptic NMDARs [37].

A previous study has shown that the decrease in the SK1 expression and intracellular S1P amount are inversely correlated with the Aβ accumulation in the brains of AD patients [38]. In addition, SK1 downregulation enhances the accumulation of Aβ, exacerbating the AD-like pathology and symptoms in the brains of AD model mice [10,11]. We showed that the decrease in endogenous S1P diminished the microglial Aβ clearance (Figure 8b), and that this change increased the number of microglia that phagocytosed damaged neurons (Figure 10). Altogether, it appears that low levels of glial S1P contribute to Aβ deposition, which is constituted of senile plaques, and that the main lesion in AD is due to an impaired Aβ clearance capacity, resulting in neuronal damage and loss.

## 5. Conclusions

This study demonstrates that a decrease in intracellular S1P inhibited the microglial Aβ uptake and increased the neuronal damage, which was accompanied by Aβ neurotoxicity. The low level of S1P exerted its effect through alterations in the glial cell functions. Although further studies are essential to clarify the detailed causal relationship between the glial S1P content and the progression of AD pathology, SK1 and S1P in glial cells may be a potential therapeutic target for AD.

## Figures and Tables

**Table 1:** Primers for real-time quantitative PCR analysis.

Gene of Interest		Primer Sequence (5′-…-3′)	Denaturation (15 s)	Annealing Extension (30 s)	Cycle	Estimated Size (bp)
PPARγ	Sense	GTACTGTCGGTTTCAGAAGTGCC	95	65	40	102
	Antisense	ATCTCCGCCAACAGCTTCTCCT				
CD36	Sense	GAACCACTGCTTTCAAAAACTGG				102
	Antisense	TGCTGTTCTTTGCCACGTCA				
18S rRNA	Sense	AGGTCTGTGATGCCCTTAGA				239
	Antisense	CCATCCAATCGGTAGTAGCG				

## Data Availability

The original contributions presented in the study are included in the article/Supplementary Material, further inquiries can be directed to the corresponding author.

## Supplementary Materials

The following supporting information can be downloaded at https://www.mdpi.com/article/10.3390/neurolint16040054/s1, Figure S1: Effects of PF-543 on cell viability in LPS-treated SH-SY5Y neuroblastoma.
