# Supplementary material for: Inhibition of Sphingosine Kinase 1 Reduces Sphingosine-1-Phosphate and Exacerbates Amyloid-Beta-Induced Neuronal Cell Death in Mixed-Glial-Cell Culture
Source: Neurol Int. 2024 Jul 4;16(4):709–30. doi: 10.3390/neurolint16040054 (PMC11270188; doi:10.3390/neurolint16040054)
Supplement: Supplementary file 1 [file neurolint-16-00054-s001.zip › neurolint-3013739-supplementary.pdf]

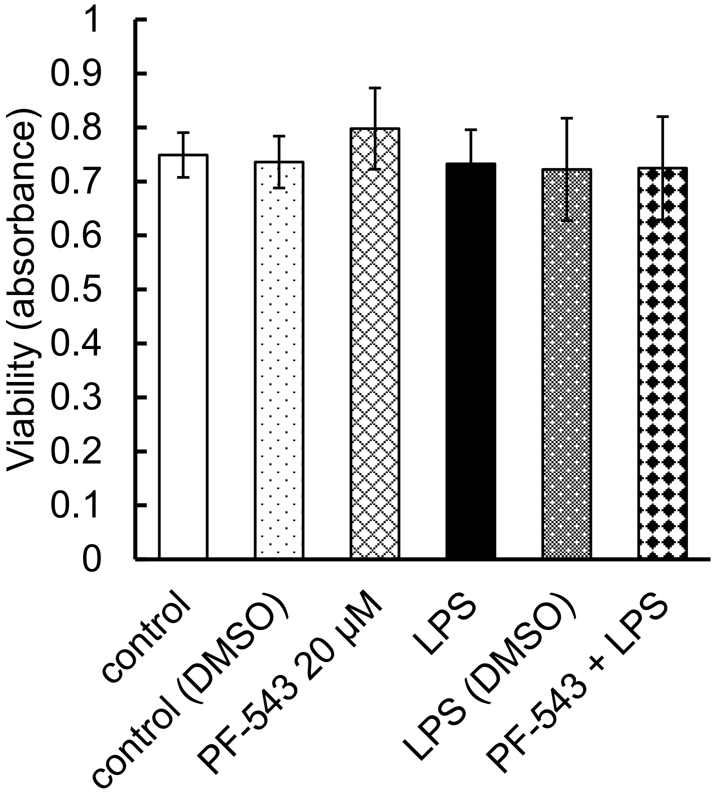

Figure S1. Effects of PF-543 on cell viability in LPS-treated SH-SY5Y neuroblastoma. SH-SY5Y cells were treated with 10 ng/mL LPS with or without 20 μM of PF-543 for 24 h. Cell viability was assessed by MTT assay.
